# Typhlitis

**Published:** 1890-04-12

**Authors:** 


### The Practitioner's Mirror and Retrospect.

[*The Editor will be glad to receive offers of co-operation and contributions from members of the profession. There is no doubt that much valuable material full of interest and instruction is at present lost to science. This is largely so in the case of* (1) *the younger and less-known members of the hospital staff* (2) *those connected with cottage hospitals, and* (3) *the great body of medical practitioners not attached to any institution. The co-operation of all is cordially invited to enable the Editor to make* The Hospital *of the utmost possible use and value to the profession. Specialists willing to review books on their special subjects are invited to communicate this fact at once. All letters should be addressed to* The Editor, The Lodge, Porchester Square, London, W.]

### MEDICINE.

### TYPHLITIS.

The *Birmingham Medical Review* contains an article by Mr. Lawson Tait on the surgical treatment of typhlitis. His experience of the surgical treatment of typhlitis now extends to 29 cases; and from that experience Mr. Tait comes to the conclusion that the earlier operative interference is employed the better for the patient. Even in cases where resolution is likely or possible, the operation can do no harm, while the likelihood of the disease ending in resolution is small. It is assumed that cases end without suppuration, but it is extremely probable that they do not do so, but that a purulent focus is left as the centre of future disturbance. The fact in support of this statement is to be found in the frequency of successive attacks of inflammation of the cœcum. The peculiar features of the disease are fixed local signs, occurring without obvious cause. The localised fulness and tension increase and extend to the contiguous parts of the abdomen, while the tenderness over the cœcum exceeds that of peritonitis. Constipation is a marked feature, but vomiting not so constant. A favourable termination cannot be counted on until the bowels begin to move. And if life be prolonged a fæcal abscess may form and make its way to the surface. Mr. Tait desires to discard such terms as perityphlitis or appendicitis, for wherever the disease commences it is impossible to distinguish by the symptoms. Mr. Tait details various cases, of which we notice the following :—A boy had the characteristic symptoms of typhlitis. On the third day the swelling was opened freely over the line of the greatest prominence. There was no pus, but relief of all symptoms was immediate, and in ten days the boy was going about. A collier had characteristic typhlitis. The patient refused operation till there was cracking under the skin. A four-inch incision was then made. This ended in fæcal fistula, from which eventually the patient recovered. Mr. Tait attributes some sloughing which occurred to the use of carbolic acid and oil. Now, notwithstanding these and other successful cases detailed by Mr. Tait we doubt if operative procedure should be had recourse to quite so early as Mr. Tait recommends, although, indeed, his practice is in accordance with that of some of the American surgeons. Still, we think that every case of inflammation of the cæcum does not end in suppuration, and that repeated attacks may occur without suppuration. Neither do we think that the operation is quite so simple and harmless as represented. In Mr. Tait's hands a patient would have the best possible chance, but every surgeon is not so conversant with abdominal surgery as Mr. Tait!

